# Supplementary material for: Inhibitory effects of black cumin on the formation of heterocyclic aromatic amines in meatball
Source: PLoS One. 2019 Aug 27;14(8):e0221680. doi: 10.1371/journal.pone.0221680 (PMC6711510; doi:10.1371/journal.pone.0221680)
Supplement: Attachment — Submitted filename: report.pdf [file pone.0221680.s001.pdf]

The paper is very interesting. From chemical point of view, there is nothing new, just another scenario of *Maillard* reaction. The data are brief and clear. The paper is well written. And it includes the relevant literature. It seems from the data presented that the black cumin can prevent the formation of genotoxic mutagenic amines to some extent. Taking into account nutritional aspects, it is a valuable finding. Therefore, I recommend the present study to be published as it is.
